# MOF Material-Derived Bimetallic Sulfide Co_x_Ni_y_S for Electrocatalytic Oxidation of 5-Hydroxymethylfurfural

**DOI:** 10.3390/nano13162318

**Published:** 2023-08-12

**Authors:** Cong Guo, Yunying Huo, Qiao Zhang, Kai Wan, Guangxing Yang, Zhiting Liu, Feng Peng

**Affiliations:** 1 School of Chemistry and Chemical Engineering, Guangzhou University, Guangzhou 510006, Chinayanggx@gzhu.edu.cn (G.Y.); liuzt@gzhu.edu.cn (Z.L.); 2 School of Chemistry and Chemical Engineering, South China University of Technology, Guangzhou 510640, China

**Keywords:** 5-hydroxymethylfurfural oxidation, electrocatalysis, biomass conversion, 2,5-furandicarboxylic acid, MOF-derived electrocatalyst

## Abstract

The electrocatalytic conversion of biomass into high-value-added chemicals is one of the effective methods of green chemistry. Conventional metal catalysts have disadvantages, such as low atomic utilization and small surface areas. Catalyst materials derived from metal–organic frameworks (MOFs) have received much attention due to their unique physicochemical properties. Here, an MOF-derived non-precious metal Co_x_Ni_y_S electrocatalyst was applied to the oxidation of biomass-derivative 5-hydroxymethylfurfural (HMF). The HMF oxidation reaction activities were modulated by regulating the content of Co and Ni bimetals, showing a volcano curve with an increasing proportion of Co. When the Co:Ni ratio was 2:1, the HMF conversion rate reached 84.5%, and the yield of the main product, 2,5-furandicarboxylic acid (FDCA), was 54%. The XPS results showed that the presence of high-valent nickel species after electrolysis, which further proved the existence and reactivity of NiOOH, as well as the synergistic effect of Co and Ni promoted the conversion of HMF. Increasing the content of Ni could increase the activity of HMF electrochemical oxidation, and increasing the content of Co could reduce the increase in the anodic current. This study has important significance for designing better HMF electrochemical catalysts in the future.

## 1. Introduction

Biomass is a sustainable and renewable non-fossil carbon resource that can be used as a fuel or converted into high-value-added fine chemicals, and this makes it a promising renewable energy source to replace existing fossil fuels [1,2,3]. The value of biomass can be maximized by converting it into high-value-added products or intermediates for the synthesis of fine chemicals rather than using it directly as fuel [4]. As a representative platform molecule for biomass derivatives, HMF is generated from hexose or pentose sugars present in lignocellulosic hydrolysates and can be further derived into a range of value-added chemicals after oxidation [5]. Among its oxidation products, 2,5-furandicarboxylic acid (FDCA) is considered to be a promising alternative to terephthalic acid for the production of glycol furan dicarboxylate (PEF, polyethylene high foam insulation), replacing the petroleum derivative polyethylene terephthalate (PET, polyester resin for fibers, films and plastics) [6,7,8]. However, conventional thermochemical HMF oxidation to FDCA usually requires reactions under demanding conditions (high alkali concentration; temperatures above 100 °C; up to 10 bar O_2_ or 40 bar air, etc.) [8,9]. In contrast to the conventional heterogeneous catalytic aerobic oxidation of HMF, electrochemical HMF oxidation has gradually received a great deal of attention due to its mild operating conditions and controlled selectivity in recent years [10]. 

In recent years, metal–organic frameworks (MOFs) have emerged as promising platform coordination compounds with a variety of special properties, such as high porosity and structural diversity [11,12,13,14,15]. Meanwhile, transition metal sulfides (TMSs) are promising catalysts due to their unique physicochemical properties, such as various crystal structures, different crystalline phases, adjustable band positions, and band gaps [16,17,18]. TMSs derived from metal–organic frameworks have attracted much attention in the field of energy conversion, and they are ideal materials for the preparation of metal compounds such as metal oxides, metal sulfides, and metal phosphides due to the presence of multiple metal ions in MOFs [19,20,21,22]. The derivatives from the MOF materials showed good stability, electrical conductivity, and highly dispersed active sites [23,24,25]. Transition metal-supported catalysts exhibit satisfactory catalytic activity for electrochemical reactions such as CO_2_ reduction, oxygen reduction reactions (ORRs), and hydrogen evolution reactions (HERs), but their uncontrolled active sites are particularly prone to deactivation [26,27,28,29]. Therefore, the development of MOF materials to regulate the reactive active sites has attracted much attention.

To date, various electrocatalysts for the oxidation of HMF have been developed, including noble metals (e.g., PtRu [30] and PdAu [31] alloys), non-precious metals (e.g., Ni_2_P [32], Ni_2_S_3_ [33], Ni_3_N [34], NiB_x_ [35], NiCoFe-LDHs [36], etc.), and non-metallic catalysts (e.g., B/N-C [37]). Then, the transition metal bimetallic catalysts were found to have great potential for HMF oxidation reactions. For example, Fe catalysts have low electrocatalytic HMF oxidation activities, but Menezes et al. found that Sn, Si, and Fe intermetallic compounds (IMC) with ordered structures and high conductivity had high efficiency for the HMF electrochemical oxidation reaction [38]. Han et al. found that there was a synergistic effect between Ni and Co for the HMF electrochemical oxidation reaction by comparing the catalytic activities of NiO, NiCo_3−x_O_4_, and Co_3_O_4_ [39].

Meanwhile, the coordination environment is also a key point for improving the electrocatalytic activity of HMF oxidation. The orbital hybridization can be tuned by changing the coordination atom species of the central active metal; thus, the electronic structure will be subtly modulated. Up to now, various Ni or Co based candidates, including nitride, sulfide, phosphide, selenide, and boride, have shown promising performances. Li et al. prepared Co-doped 2D MOFs NiCoBDC with a high FDCA yield of 99% and a Faradaic efficiency of 78.8% at 1.55 V vs. RHE [12]. Qi et al. fabricated a facile solvothermal method to prepare ternary (Co, Ni and Fe) MOF nanoarrays onto Ni foam, which had highly efficient HMF electrooxidation activities with a high FDCA yield of 99.76% and a Faradaic efficiency close to 100% [40]. Zeng et al. demonstrated that the cobalt and nickel bimetallic MOF-derived NiCo–S catalyst had high HMF electrooxidation activity. The Co site promotes the transformation of the CH=O group to the carboxyl group owing to its strong diatomic adsorption (O and C atoms) on the CH=O group, and the Ni site further accelerates the reaction rate for FDCA production [41].

To achieve superior HMFOR electrocatalytic activity, the construction of dual sites to form synergism between two metal centers is the effective strategy. Here, the transition metal sulfide Co_x_Ni_y_S was prepared by pyrolysis of bimetallic Co_x_Ni_y_-MOF, and the feasibility of HMF oxidation with this MOF-derived catalyst was explored. It was found that the electrochemical HMF oxidation performance of Co_x_Ni_y_S showed a volcano curve with an increasing proportion of Co. The appropriate doping of Co metal reduced the overpotential of the HMF electrooxidation reaction and promoted the generation of high-valent nickel species, and the Co and Ni in the Co_2_NiS catalyst synergistically promoted the oxidation of HMF. The transition metal MOF derivatives were used as anodes to construct the oxidation system of HMF, which made it possible to convert HMF into high-value chemicals. This work is instructive for the synthesis of novel MOF material derivatives for biomass conversion applications.

## 2. Experimental Section

### 2.1. Chemicals

Nickel nitrate hexahydrate (Ni(NO_3_)_2_·6H_2_O, AR, 98%), cobalt nitrate hexahydrate (Co(NO_3_)_2_·6H_2_O, AR, 99%), 1,3,5-benzenetricarboxylic acid (>98%), N, N-dimethylformamide (DMF, AR, 99.5%), sulfur sublimed (>99.5%), 4,4′-bipyridine (C_10_H_8_N_2_, AR, 98%), ammonium formate (AR, 98%), 5-(hydroxymethyl)furfural (HMF, AR, 98%), 2,5-furandicarboxylic acid (FDCA, AR, 98%), 5-formyl-2-furancarboxylic acid (FFCA, AR, 98%), 5-hydroxymethyl-2-furancarboxylic acid (HMFCA, AR, 98%), 2,5-diformylfuran (DFF, AR, 98%), ethanol (EtOH, AR, 99%), and methanol (CH_3_OH, AR, 99%) were purchased from Aladdin Chemicals Co., Ltd. (Shanghai, China). Deionized water was used in all experiments.

### 2.2. Preparation of Materials

**Figure 1:**
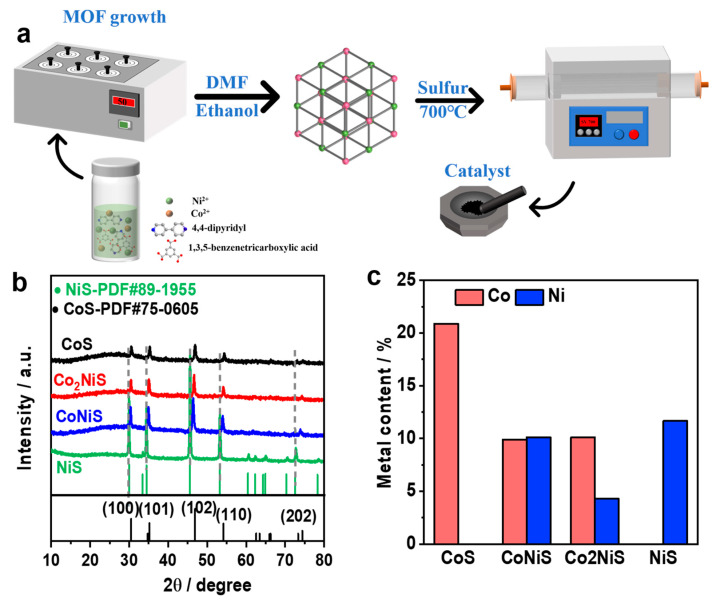
(**a**) A schematic diagram of the preparation of the catalysts. (**b**) XRD patterns of the obtained CoS, Co_2_NiS, CoNiS, and NiS. (**c**) The ICP results of the metal content (wt. %) of the catalysts.

**Preparation of Co_x_Ni_y_-MOF:** For this experiment, 2.910 g Co(NO_3_)_2_-6H_2_O, 0.582 g Ni(NO_3_)_2_-6H_2_O, 0.844 g 1,3,5-benzenetricarboxylic acid, and 0.768 g 4,4-bipyridine were dissolved in 80 mL N, N-dimethylformamide and heated to 50 °C for 72 h. The products, named Co_2_Ni-MOF, were then washed with DMF and ethanol and dried at 60 °C for 12 h in the air. CoNi-MOF was obtained from 2.328 g Co(NO_3_)_2_∙6H_2_O and 1.164 g Ni(NO_3_)_2_∙6H_2_O. Co-MOF and Ni-MOF were also prepared via the same procedure, except that only one metal salt was used (all of the above nomenclatures are based on the metal content ratio) (Figure 1c).

**Synthesis of the Co_x_Ni_y_S catalyst:** In this step, 0.72 g metal–MOF material and 1.92 g sublimated sulfur taken as the sulfur source were mixed together, then loaded into a porcelain boat and transferred to a tube furnace, heated to 700 °C under a nitrogen atmosphere with a heating rate of 5 °C min^−1^, and held for 2 h to finally obtain the metal sulfide. The samples were called CoS, CoNiS, Co_2_NiS, and NiS. 

### 2.3. Materials Characterizations

The crystal structure was characterized by an X-ray diffractometer (XRD) (PW3040/60 from PANalytical, Almelo, The Netherlands). The test conditions were as follows: Cu Kα-rays, λ = 0.15418 nm, maximum voltage of 60 kV, maximum current of 55 mA, scanning at 10 °C min^−1^, and a scan range of 5–80°. Transmission electron microscope (TEM) images were taken using a JEOL JEM-2100F at 200 kV. Scanning electron microscope (SEM) images were taken using a JEOL JSM-7001F cold field emission scanning electron microscope to observe the morphology and structure of the catalyst. The metal content in the metal sulfides was analyzed by inductively coupled plasma emission spectrometry (ICP-OES, Agilent 720ES, Agilent, Amstelveen, The Netherlands). The specific surface area was measured using the Brunauer–Emmett–Teller (BET) method based on the N_2_ adsorption and desorption isotherms at 77 K on a Tristar II I3020 from Micromeritics Instruments. The Barrett–Joyner–Halenda (BJH) pore size model was used to calculate the average pore diameter according to the desorption branch of the isotherms. ICP-AES analyses were carried out via inductively coupled plasma atomic spectrometry (ICPS-8100 Shimadzu, Kyoto, Japan). X-ray photoelectron spectroscopy (XPS) was performed using a Thermo Scientific K Alpha Nexsa model X-ray photoelectron spectrometer (Thermo Scientific, Waltham, MA, USA). The C1s peak was calibrated by 284.8 eV, and split-peak fits were performed using Advantage software5.5.

### 2.4. Electrochemical Measurements

Electrochemical measurements were carried out with a CHI 760E electrochemical analyzer, and all tests were carried out in a single-chamber, three-electrode cell under ventilated conditions unless otherwise stated. The electrolytes with HMF dissolved in them were stirred in the electrochemical cell for a few minutes to ensure that the HMF molecules were adsorbed on the electrode surface adequately before the test. The working electrode was nickel foam (1 × 1 cm^2^) loaded with 5 mg of catalysts, the reference electrode was Hg/HgO (1 M KOH), and Pt sheets were used as counter electrodes. The nickel foam was cut and sonicated with ethanol, acetone, and 4 M HCl for 10 min, respectively, then dried at 60 °C. For ink preparation, 5 mg of the catalyst was dispersed in 300 μL of ethanol, 60 μL of deionized water, and 40 μL of 5% Nafion solution; sonicated for 30 min and mixed well; then dropped onto the nickel foam and dried at room temperature.

Linear scanning voltammetry (LSV) was carried out in 0.1 M KOH or in the presence of 10 mM HMF in 0.1 M KOH at a rate of 10 mV s^−1^ at room temperature. The potential was converted to reversible hydrogen potential using E_RHE_ = E_Hg/HgO_ + 0.0591 × pH + 0.098 V. Constant potential electrolysis (chronoamperometric measurements) was tested in a 3H type. In the cell, the electrolyte consisted of 0.1 M KOH solution containing 10 mM HMF. The potential was 1.45V (vs. RHE), and a change in current density with time was observed. The cyclic voltammetry tests (CV) were conducted in 0.1 M KOH with 10 mM HMF electrolyte solution at different scanning rates (10, 20, 30, 40, and 50 mV s^−1^) in a non-Faradaic potential region (0.41–0.61 V vs. RHE). The electrochemical activity specific surface area (ECSA) was evaluated by electric double layer capacitance (*C_dl_*) with the following formula:

$$ECSA=\frac{C_{dl}}{C_{s}}$$

The difference between positive and negative currents at different sweep rates at the intermediate potential region was plotted, and the value of *C_dl_* was half of the slope of linear fitting. *C_s_* was the capacitance measured from the ideal electrolyte condition or ideally smooth surface catalyst, and here, the C_s_ value used the typical value of 0.04 mF cm^−2^.

### 2.5. Product Analysis

Sample solutions of 20 μL were taken out of the anode chamber, diluted with 980 μL of water, and then detected by a liquid chromatography (HPLC) instrument equipped with a UV-Vis detector at 265 nm. A 4.6 nm × 250 nm ZORBAX Eclipse XDB 5 μm C18 column was used to separate the reactants and the products. The mobile phase A was methanol, the mobile phase B was 5 mM ammonium formate aqueous solution, the ratio of A:B was 25:75, and the flow rate was 0.6 mL min^−1^ at 40 °C for 8 min.

HMF conversion, FDCA yield, selectivity, and Faraday efficiency (FE) were calculated using Equations (1)–(4), respectively.

(1) $$\mathrm{HMF}\;\mathrm{conversion}\;(\%)=[n\left(\mathrm{HMF}\;\mathrm{consumed}\right)/n\left(\mathrm{HMF}\;\mathrm{initial}\right)]\;\times 100$$

(2) $$\mathrm{Yield}\;\mathrm{of}\;\mathrm{product}\;\left(\%\right)=[n\left(\mathrm{product}\;\mathrm{formed}\right)/n\left(\mathrm{HMF}\;\mathrm{initial}\right)]\;\times 100$$

(3) $$\mathrm{Selectivity}\;(\%)=[n\left(\mathrm{product}\;\mathrm{formed}\right)/n\left(\mathrm{HMF}\;\mathrm{consumed}\right)]\;\times 100$$

(4) $$\mathrm{Faradaic}\;\mathrm{efficiency}\;(\%)=[n\left(\mathrm{product}\;\mathrm{formed}\right)/\left(\frac{\mathrm{Charge}}{z\;\times F})\right]\;\times 100$$

where F is the Faraday constant (96,485 C mol^−1^) and n is the mole of the reactant, calculated from the concentration measured by HPLC. The *z* was the number of electrons transferred by the oxidation of HMF to the corresponding product.

## 3. Results and Discussion

The Co_x_Ni_y_S samples were synthesized by a simple pyrolysis method using bimetallic MOF as the precursor, and a schematic diagram of the catalyst preparation process is shown in Figure 1a. The Co_x_Ni_y_-MOF precursor was synthesized via a solvothermal reaction with cobalt, and nickel salts and organic matter were combined in N,N-dimethylformamide (DMF) at 50 °C for 72 h. The X-ray diffraction pattern in Figure 1b showed a cobalt–nickel-doped metal sulfide with peaks at 29.9°, 34.4°, 45.5°, and 53.2°. The diffraction peaks at 29.9°, 34.4°, 45.5°, 53.2°, and 72.6° corresponded to the (100), (101), (102), (110), and (202) planes of NiS (PDF#89-1955), respectively, and the diffraction peaks at 30.4°, 34.7°, 46.8°, 54.1°, and 74.4° corresponded to the (100), (101), (102), and (110) planes of CoS (PDF#75-0605), respectively. Co_x_Ni_y_S showed the same diffraction peaks as the monometallic cobalt sulfide and nickel sulfide, but as the proportion of nickel increased, the diffraction peaks of the Co_x_Ni_y_S catalysts slightly shifted towards lower angles, further indicating that the Co and Ni metals were co-doped and revealing the existence of interactions between the nickel–cobalt elements in the Co_x_Ni_y_S catalysts. Figure 1c presents the metal content of the catalysts from inductively coupled plasma emission spectrometry (ICP-OES), and the results showed that the metal contents of Co and Ni were 9.9% and 10.12% and 10.12% and 4.28% for the CoNiS and Co_2_NiS catalysts, respectively.

**Figure 2:**
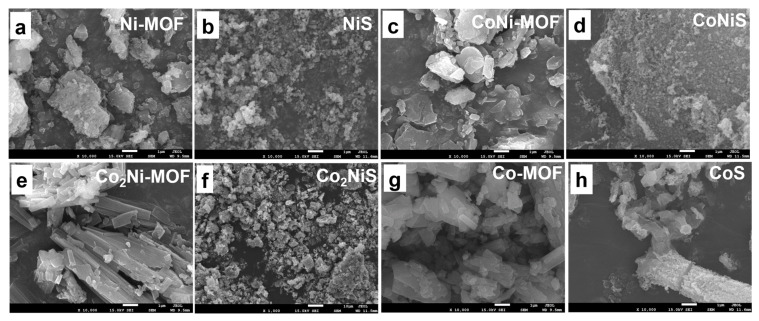
SEM images of (**a**) Ni-MOF, (**b**) NiS, (**c**) CoNi-MOF, (**d**) CoNiS, (**e**) Co_2_Ni-MOF, (**f**) Co_2_NiS, (**g**) Co-MOF, and (**h**) CoS.

Figure 2 shows the SEM images of the Co_x_Ni_y_-MOF and the Co_x_Ni_y_S. The Co-MOF precursors exhibited irregular square-shaped morphology, and the Ni-MOF exhibited a flaky structure. The CoNi-MOF materials showed a mixture of small flakes and squares, and with the proportion of cobalt being increased, the Co_2_Ni-MOF showed an obvious striped structure (Figure 2e). After the pyrolysis of Co_x_Ni_y_-MOF with sulfur powder at 700 °C under nitrogen, the sulfur atoms diffused into the metal sites and formed metal sulfides. The samples showed loose and porous particles, indicating an increased specific surface area.

**Figure 3:**
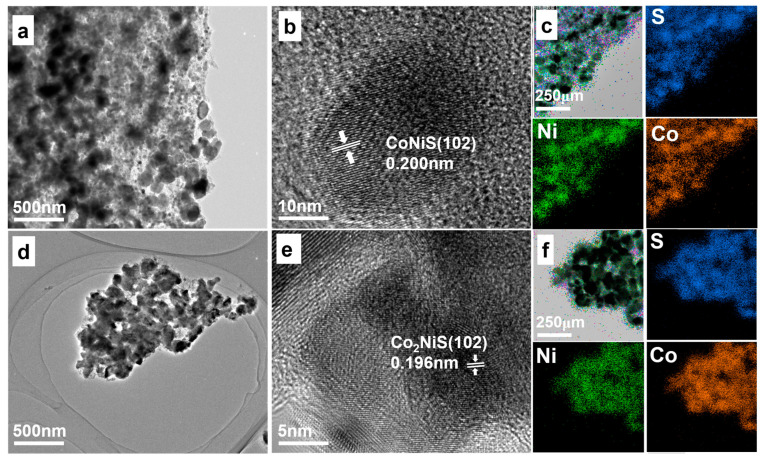
(**a**,**b**) TEM images of CoNiS. (**c**) Element mapping images of CoNiS. (**d**,**e**) TEM images of Co_2_NiS. (**f**) Element mapping images of Co_2_NiS.

The Co_x_Ni_y_S nanomaterials with different Ni and Co ratios, characterized by transmission electron microscopy (TEM), are shown in Figure 3. Figure 3a,d show the TEM images of CoNiS and Co_2_NiS. Figure 3b,e show the HRTEM images of CoNiS and Co_2_NiS. The results indicate that CoNiS and Co_2_NiS were well crystalline. The lattice spacing of 0.200 nm corresponds to the (102) crystal plane of the CoNiS catalyst, and the lattice spacing of 0.196 nm corresponds to the (102) crystal plane of the Co_2_NiS catalyst. Energy-dispersive X-ray spectroscopy (EDS) was carried out as shown in Figure 3c,f. The distributions of the Co, Ni, and S elements almost overlapped, indicating that the sulfide was successfully homogeneously coupled with the cobalt and nickel bimetals. The (102) lattice spacing of the Co_x_Ni_y_S catalyst gradually decreased with the increase in Co proportion, with NiS (0.205 nm) > CoNiS (0.200 nm) > Co_2_NiS (0.196 nm) > CoS (0.193 nm), which was consistent with the XRD results (Figure 1b). The crystal plane angle shifted towards the positive direction, corresponding to a gradual decrease in the lattice spacing with the increasing proportion of cobalt.

Figure S1a,c shows that both NiS and CoS had encapsulated structures with the carbon layer. Figure S1b,d shows the distinct lattice striations observed for NiS and CoS, indicating good crystallinity with a lattice spacing of 0.205 nm, representing the (102) crystal plane for NiS, and 0.193 nm and 0.293 nm lattice spacing for CoS, representing its (102) and (100) crystal planes, respectively.

**Figure 4:**
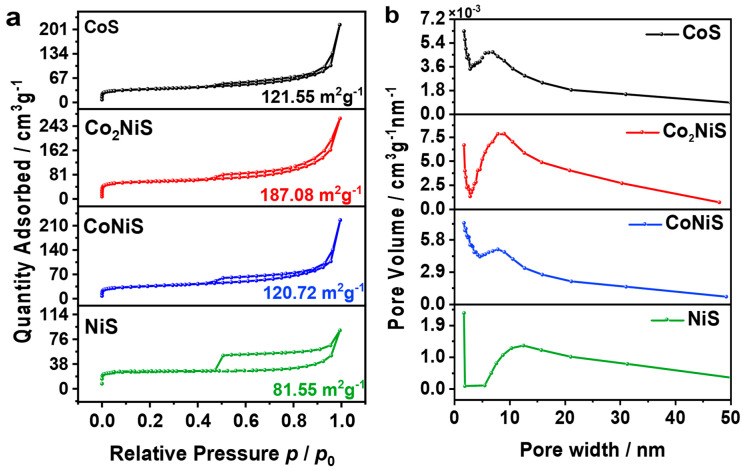
(**a**) Nitrogen adsorption and desorption curves. (**b**) The corresponding pore size distribution.

Figure 4a shows the N_2_ adsorption/desorption curves of the Co_x_Ni_y_S catalysts, and they all exhibited type IV isotherms. The specific surface areas of the NiS, CoNiS, Co_2_NiS, and CoS catalysts were 81.55, 120.72, 187.08, and 121.55 m^2^ g^−1^, respectively, showing a volcano-type curve as the content of Co increased. The specific surface area of Co_2_NiS was the largest. This may indicate that when the metal content ratio of Co:Ni reached 2:1, the Co_2_NiS catalyst provided more active sites. Figure 4b shows the pore size distribution of the Co_x_Ni_y_S catalyst with a mesoporous structure (2–50 nm).

**Figure 5:**
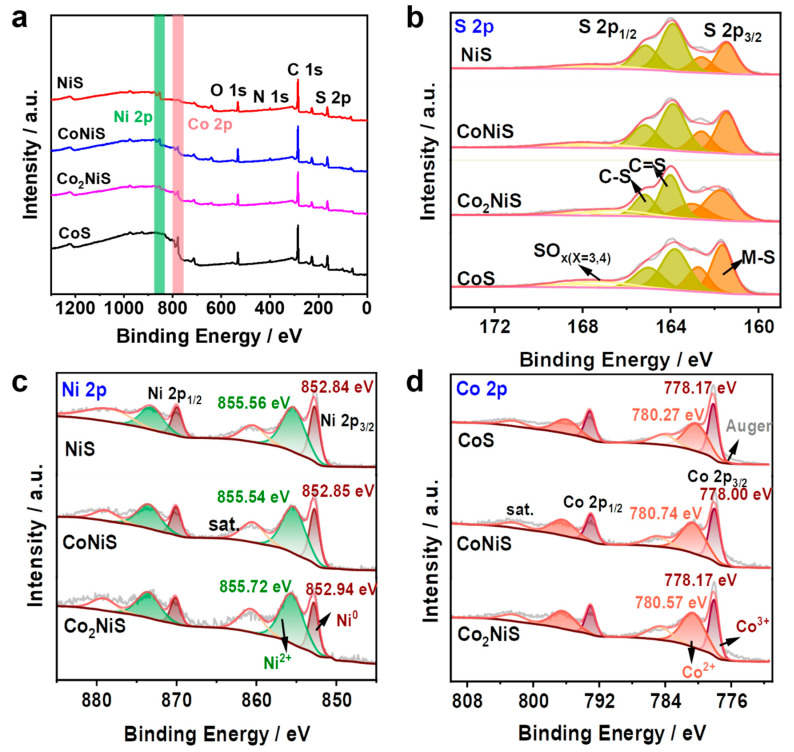
(**a**–**d**) XPS survey spectra and S 2*p*, Ni 2*p*, and Co 2*p* spectra of CoS, NiS, CoNiS, and Co_2_NiS.

The elemental composition and electronic interactions on the surfaces of the bimetallic sulfides were investigated by X-ray photoelectron spectroscopy (XPS). As shown in Figure 5a, the full spectra showed the presence of several elements of Ni, Co, O, S, N, and C (Figure S2). The oxygen possibly originated from the surface oxidation of nitrogen-doped carbon metal sulfides and nitrogen oxides, with the peaks at 531.43 eV and 533.0 eV corresponding to oxygen species in metal–oxygen bonds and OH^-^ functional groups, respectively (Figure S2b) [42,43]. Figure 5b shows the S 2*p* spectrum containing S 2*p*_3/2_ and S 2*p*_1/2_, which can be attributed to the sulfur ion in the metal ion coordination [44,45]. Figure 5c shows the Ni 2*p* for NiS, CoNiS, and Co_2_NiS. The Ni 2*p*_3/2_ peaks at 852.84 eV and 855.56 eV represent the metallic Ni and Ni^2+^ species, and the peaks at 860.45 eV and 878.04 eV indicate the satellite peaks of metallic Ni for NiS [46,47]. Figure 5d shows the Co 2*p* for NiS, CoNiS, and Co_2_NiS. The Co 2*p*_3/2_ peaks at 780.35 eV and 778.15 eV corresponded to Co^2+^ and Co^3+^, respectively, and the peaks at 784.72 eV and 802.81 eV were characteristic of the metallic Co. Since the Co 2*p* region overlapped with some of Co LMM oscillations, the Co LMM oscillation peak likely occurred at around 776 eV [48,49]. For the Co 2*p* orbitals, as the proportion of Co increased, the binding energy of Co^2+^ shifted negatively by 0.17 eV and 0.47 eV for Co_2_NiS and CoS, respectively, while the binding energy of Ni^2+^ shifted positively by 0.18 eV for Co_2_NiS [50,51].

**Figure 6:**
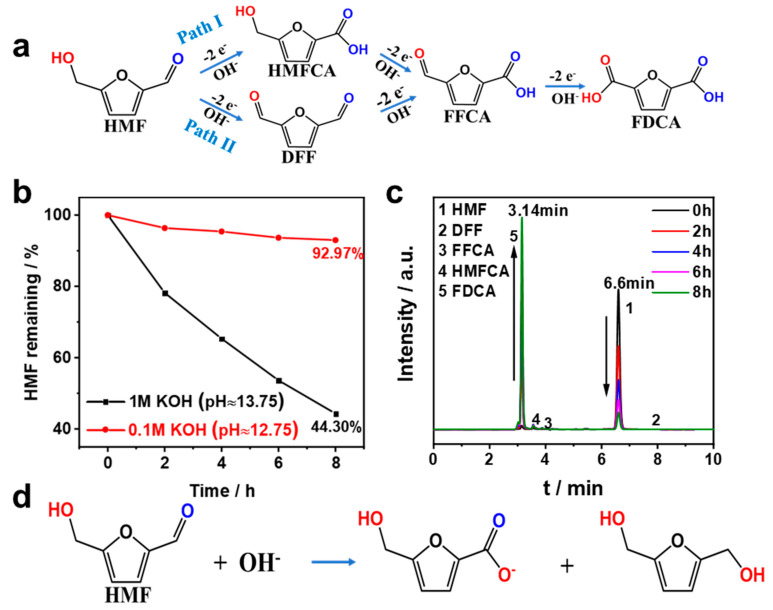
(**a**) Reaction pathways of HMF oxidation. (**b**) Stability test of 10 mM HMF in different electrolytes. (**c**) HPLC chromatograms at various electrolysis time points. (**d**) Possible self-degradation mechanism via the Cannizzaro reaction.

In general, there were two possible pathways of HMF to FDCA, as shown in Figure 6a. The aldehyde group of HMF was first oxidized to a carboxyl group to form 5-hydroxymethyl-2-furancarboxylic acid (HMFCA), and the other pathway involved the oxidation of the hydroxyl group to an aldehyde group to form 2,5-dicarboxyfuran (DFF). Then, the HMFCA and DFF were further oxidized to 5-formyl-2-furancarboxylic acid (FFCA) and, finally, FDCA [32,33]. The anodic half-cell reaction may occur as follows:

HMF + 2OH^−^ → DFF + 2H_2_O + 2e^−^

or HMF + 2OH^−^ → HMFCA + 2H_2_O + 2e^−^

DFF + 2OH^−^ → FFCA + 2H_2_O + 2e^−^

or HMFCA + 2OH^−^ → FFCA + 2H_2_O + 2e^−^

FFCA + 2OH^−^ → FDCA + H_2_O + 2e^−^

The total cathodic half-cell reaction may occur as follows:

6H_2_O + 6e^−^ → 6OH^−^ + 3H_2_

The electrochemical oxidation of HMF usually occurred under alkaline conditions, but we found that HMF was unstable at high pH levels. Figure 6b,c show the time-concentration profiles and HPLC chromatogram profiles of 10 mM HMF in KOH solution. The results showed that less than 50% of HMF remained after 8 h in 1 M KOH solution without any treatment, and the HMF remained around 90% in a 0.1 M KOH solution. This phenomenon was mainly attributed to the fact that the aromatic aldehyde without α-hydrogen atoms of HMF undergoes its redox via the Cannizzaro reaction (Figure 6d) at high concentrations of OH^−^, and therefore, the electrochemical oxidation test of HMF should be carried out at lower alkaline conditions [12]. Thus, 0.1 M KOH solution was chosen for the electrochemical oxidation of HMF in this experiment [13].

To investigate the relationship between HMF oxidation activities and the electrocatalyst structure, linear sweep voltammetry (LSV) scans were conducted. The electrochemical properties of Co_x_Ni_y_S in the 0.1 M KOH electrolyte with the addition of 10 mM HMF were examined in the range of 0.9 to 1.9 V (vs. RHE).

**Figure 7:**
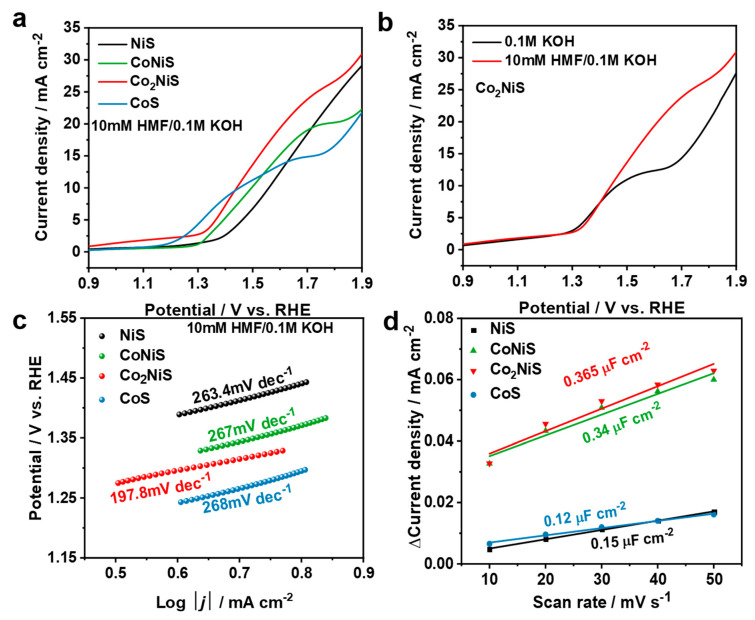
(**a**) LSV curves of NiS, CoNiS, Co_2_NiS, and CoS in 0.1 M KOH with 10 mM HMF; (**b**) LSV curves of Co_2_NiS in 0.1 M KOH and 10 mM HMF in 0.1 M KOH; (**c**) Tafel plots of different catalysts in 0.1 M KOH at a scan rate of 10 mV s^−1^; (**d**) electrical double-layer capacitance of the catalysts derived from the current density vs. scan rate.

As shown in Figure 7a, the increase in the anodic current of NiS was 1.35 V (vs. RHE), while the increase in the anodic currents of CoNiS (1.25 V), Co_2_NiS (1.28 V), and CoS (1.17 V) gradually moved towards lower potentials as the proportion of Co increased, indicating that the doping of cobalt lessened the increase in the anodic current. As shown in Figure 7b, when 10 mM HMF was added to the solution, the increase in the anodic current shifted slightly towards a lower potential, and the current density increased. Figure 7c shows the Tafel curves for all catalysts in the solution containing HMF, where the Tafel slopes for NiS, CoNiS, Co_2_NiS, and CoS were 263.4, 267, 197.8, and 268 mV dec^−1^, respectively. The Tafel slope for Co_2_NiS was the lowest, and its value was lower than in the solution without HMF, indicating that the Co_2_NiS catalyst showed a superior kinetic process for the electrooxidation of HMF [52] (Figure S3).

The electrochemical-activity-specific surface areas of all catalysts were further investigated, as shown in Figure 7d. The Co_x_Ni_y_S catalysts were subjected to CV tests at different sweep rates of 0.41–0.61 V (vs. RHE) in a 0.1 M KOH solution, which was used to calculate their bilayer capacitance (*C_dl_*) (Figure S4). The results showed a *C_dl_* of 0.15 μF cm^−2^ for NiS, 0.34 μF cm^−2^ for CoNiS, 0.365 μF cm^−2^ for Co_2_NiS, and 0.12 μF cm^−2^ for CoS. The chemically active specific surface area was usually related to the value of *C_dl_*; thus, the result suggests that compared to monometallic sulfide catalysts, bimetallic sulfides have higher electrochemically active specific surfaces, and may provide more adsorption sites for the HMFOR process.

**Figure 8:**
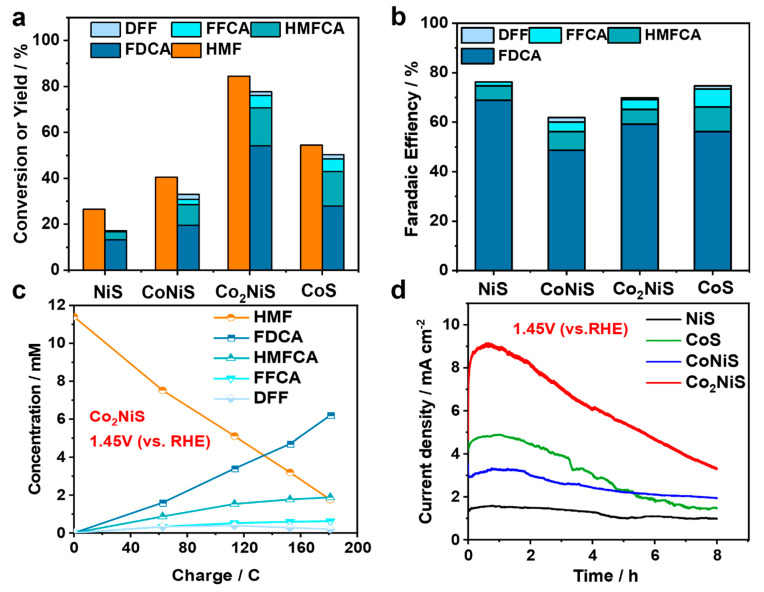
(**a**) Conversion of HMF and product yield on the different catalysts in 1.45 V (vs. RHE) for 4 h; (**b**) Faradaic efficiency of catalysts; (**c**) the concentration of substrates, intermediates, and products during HMFOR for Co_2_NiS; (**d**) the current–time transients during constant potential electrolysis at 1.45 V (vs. RHE).

Figure 8 shows the electrochemical HMF oxidation activities of the NiS, CoNiS, Co_2_NiS, and CoS catalysts. Figure 8a provides the HMF conversion and the yields of various products. The liquid chromatography (HPLC) test showed that FDCA was the main product, while 5-hydroxymethyl-2-furan carboxylic acid (HMFCA) and 2-formyl-5-furancarboxylic acid (FFCA) reaction intermediates were also found during the reaction, and a very small amount of 2,5-diformylfuran (DFF) was detected. The electrochemical oxidation performance of NiS, CoNiS, Co_2_NiS, and CoS on HMF showed a volcano curve with an increasing proportion of Co. The best performance was obtained for the Co_2_NiS electrode, which was subjected to constant potential electrolysis of 10 mM HMF and achieved an 84.5% HMF conversion rate and 54.0% FDCA yield at 1.45 V (vs. RHE) for 8 h. Figure 8b shows the Faraday efficiencies of the various products for the HMF electrocatalytic oxidation reaction by the NiS, CoNiS, Co_2_NiS, and CoS catalysts at 1.45 V (vs. RHE). The trends in Faraday efficiency were not the same as the trend in the activity of the HMF electrochemical oxidation reaction. The Co_2_NiS catalyst had the highest yield of FDCA, but lower Faraday efficiency, probably because of its high reaction current.

As shown in Figure 8c, with the increase in the transferred charge, the concentration of HMF decreased and the FDCA product was continuously generated, while the concentrations of the DFF intermediate products first increased and then decreased. In addition, Figure 8d showed the current–time curve performances of all the catalysts. For the Co_2_NiS catalysts, the current density gradually decreased with time, but its current density always remained higher than that of the CoS, CoNiS, and NiS catalysts. Table S1 demonstrates a comparison of the HMF electrochemical oxidation performances in 0.1 M KOH solution.

In summary, Co_2_NiS showed the best electrochemical oxidation activities of HMF, meaning that the electrochemical catalytic HMF performance increased as the Co content increased. The performance of pure CoS was not as good as that of Co_2_NiS. It is speculated that the doping of Ni can enhance the electro-catalytic oxidation of HMF by CoS.

**Figure 9:**
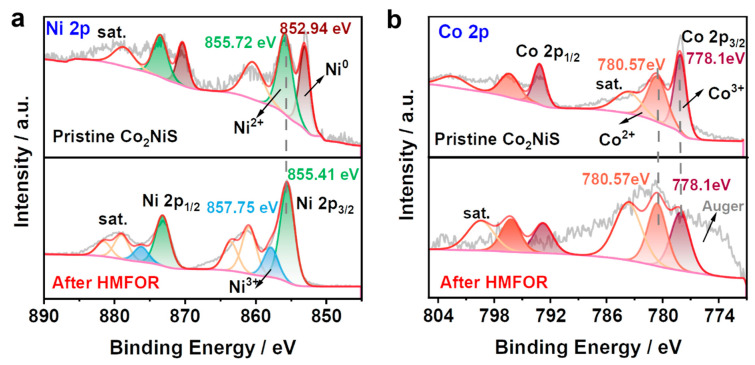
XPS spectra of (**a**) Ni 2*p* and (**b**) Co 2*p* of Co_2_NiS before and after HMFOR.

To deeply understand the relationship between the structure and activity of catalysts for HMF oxidation, the statuses of active sites should be identified. The analysis of the chemical state for Co_2_NiS before and after cyclic electrolysis, investigated by XPS spectra, is shown in Figure 9. Figure 9a,b demonstrates the peaks of Ni 2*p* and Co 2*p* of Co_2_NiS before and after HMFOR. Figure S5 shows the full spectrum and the C 1*s*, O 1*s*, and S 2*p* XPS spectra of the catalyst Co_2_NiS, respectively. As shown in Figure S5a, the peak signal of O 1*s* in the full spectrum of Co_2_NiS became relatively stronger after HMFOR. S 2*p* disappeared after the reaction, indicating that the catalyst surface changed and the elemental sulfur was dissolved into the electrolyte during electrolysis. As shown in Figure S5b, the peak of C 1*s* appears around 290 eV after HMFOR due to the element K in the KOH remaining on the catalyst surface. Figure S5c shows the split peaks of O 1*s* for Co_2_NiS before and after HMFOR. The peaks at 531.18 eV and 532.58 eV for O 1*s* before the reaction were associated with the O species in the OH^–^ functional group and chemisorbed water, and a decrease in adsorbed water could be seen after electrolysis [42,43].

The Ni 2*p* orbitals of Co_2_NiS after HMFOR are shown in Figure 9a. Compared to the pre-test Ni 2*p* orbitals, the metallic Ni peak at 852.94 eV disappeared and a peak at 857.75 eV appeared due to the Ni^3+^ species, indicating that Co_2_NiS had high-valence Ni species on the surface after HMFOR. Figure 9b shows the Co 2*p* orbital splitting of Co_2_NiS before and after HMFOR. It can be seen that the valence state of cobalt did not change significantly before and after the reaction of the catalyst, still pointing to the cobalt LMM Osher peak at around 776.0 eV, and the peaks at 778.10 eV and 780.57 eV correspond to Co^3+^ and Co^2+^, respectively [48,49]. The changes in Co species before and after the reaction were calculated, and it was found that Co^3+^/Co^2+^ was 0.67 before and 0.66 after the reaction, indicating that the ratio of Co^3+^/Co^2+^ did not change significantly after the reaction. This further demonstrates that the incorporation of Co in the Co_2_NiS catalyst promotes the production of high-valent nickel species in the electrolysis of HMF.

Thus, the mechanism of the HMF electrochemical oxidation reaction was hypothesized. When HMF was not added to the electrolyte solution, the Ni element of Co_2_NiS catalyst was able to restructure and form to Ni(OH)_2_ in the KOH solution. With the increase in potential, Ni(OH)_2_ lost electrons to form NiOOH. When HMF was added to the solution, HMF reacted quickly with NiOOH, and the NiOOH was reduced after being consumed by HMF [53,54]. The presence of cobalt can promote the formation of high-valent nickel species, and cobalt and nickel can synergistically promote the HMFOR process [41]. These results clearly demonstrate that coupling between cobalt and nickel could boost the formation of high-valence nickel species, which are considered as catalytic sites for the oxidation of HMF molecules.

## 4. Conclusions

Co_x_Ni_y_-MOF materials with different metal ratios were synthesized by a simple hydrothermal method, followed by high-temperature calcination to synthesize bimetallic sulfide Co_x_Ni_y_S catalysts for HMF electrocatalytic oxidation. TEM and XRD tests demonstrated that the Co and Ni bimetals were successfully co-doped and that the synthesized Co_2_NiS catalysts had the largest specific surface area. The electrochemical oxidation performance of Co_x_Ni_y_S on HMF showed a volcano curve with an increase in Co. The best performance was achieved on the Co_2_NiS electrode, with an 84.5% HMF conversion rate and a 54% FDCA yield. By means of an XPS test on the Co_x_Ni_y_S catalyst before and after electrolysis of HMF, the high-valent nickel species Ni^3+^ was detected on the catalyst’s surface, indicating that the presence of Co promoted the formation of this high-valent nickel species, and that Co and Ni synergistically promoted the HMFOR process.

## Data Availability

No new data were created.

## Supplementary Materials

The following supporting information can be downloaded at: https://www.mdpi.com/article/10.3390/nano13162318/s1, Figure S1. TEM images of (a,b) NiS and (c,d) CoS. Figure S2. (a) C 1*s*, (b) O 1*s* and (c) N 1*s* spectra of Co_x_Ni_y_S. Figure S3. Tafel plots of Co_2_NiS in 0.1 M KOH with and without 10 mM HMF at a scan rate of 10 mV s^−1^. Figure S4. CV of (a) NiS, (b) CoS, (c) CoNiS, and (d) Co_2_NiS in 0.1 M KOH at various scan rates for estimation of double-layer capacitance. Figure S5. (a) The XPS survey spectra, (b) C 1*s*, (c) O 1*s* and (d) S 2*p* spectra of Co_2_NiS before and after HMFOR. Table S1. Comparison of HMF oxidation performance in literature [12,31,55,56,57,58].
