# Supplementary material for: MOF Material-Derived Bimetallic Sulfide CoxNiyS for Electrocatalytic Oxidation of 5-Hydroxymethylfurfural
Source: Nanomaterials (Basel). 2023 Aug 12;13(16):2318. doi: 10.3390/nano13162318 (PMC10459279; doi:10.3390/nano13162318)
Supplement: Supplementary file 1 [file nanomaterials-13-02318-s001.zip › nanomaterials-2506871-supplementary.pdf]

Supporting Information

MOF material-derived bimetallic sulfide Co_xNi_yS for electrocatalytic oxidation of 5-hydroxymethylfurfural

Cong Guo,[a] Yunying Huo,[a] Qiao Zhang,*[a] Kai Wan,*[b] Guangxing Yang,[a] Zhiting Liu,[a] Feng Peng*[a]

[a] *School of Chemistry and Chemical Engineering, Guangzhou University, Guangzhou 510006, China*

[b] *School of Chemistry and Chemical Engineering, South China University of Technology, Guangzhou 510640, China*

* Corresponding author, Email: fpeng@gzhu.edu.cn (F. Peng);

zhangqiao@gzhu.edu.cn (Q. Zhang)

wank@scut.edu.cn (K. Wan)

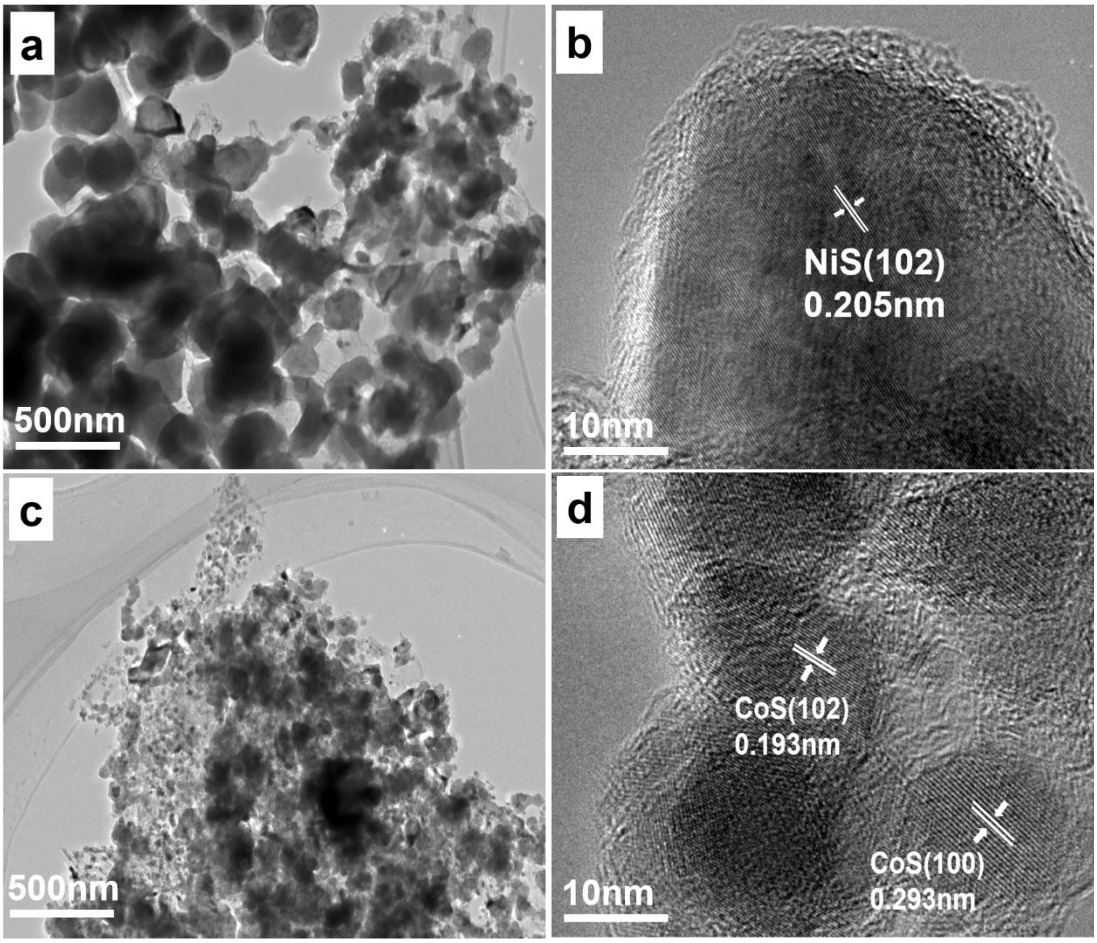

Figure S1 TEM images of (a, b) NiS and (c, d) CoS.

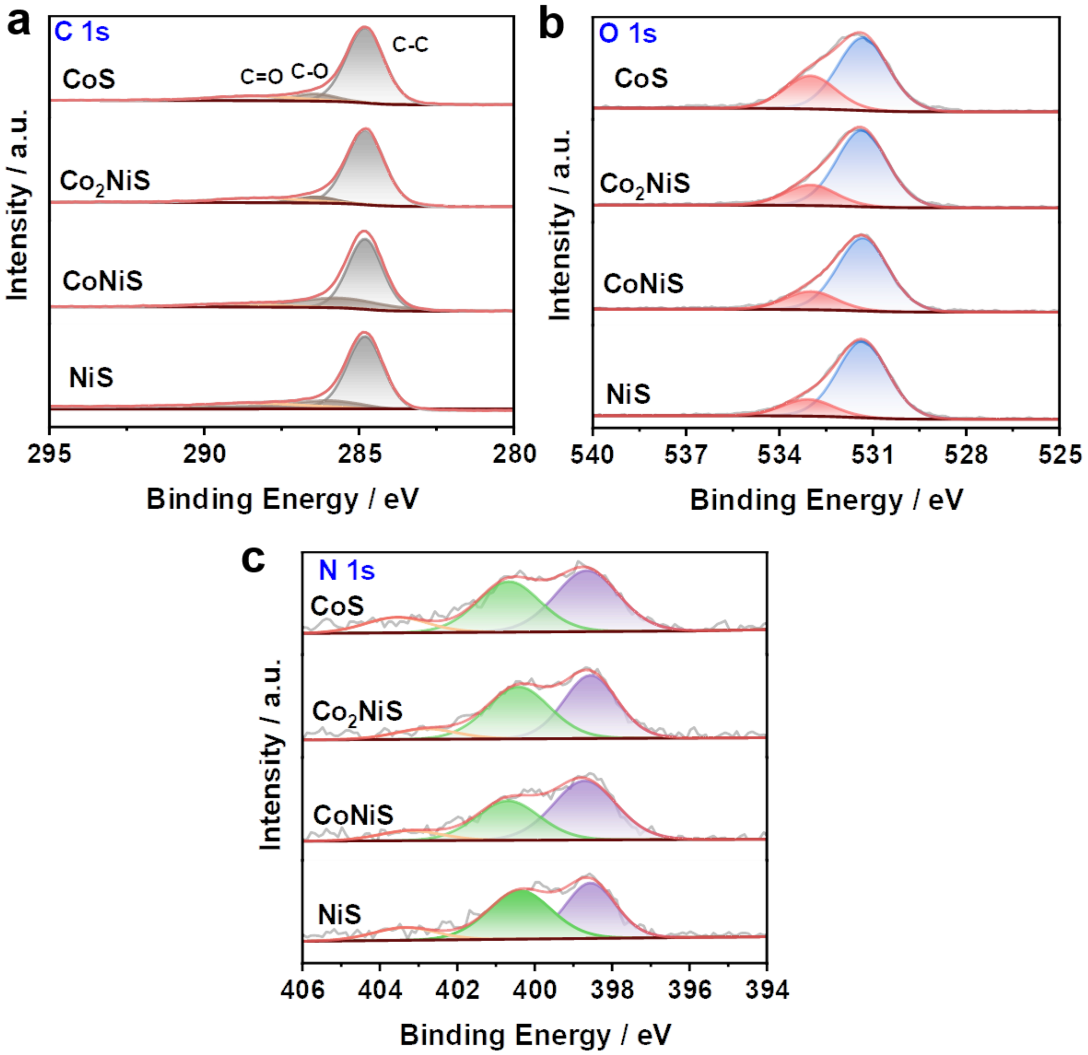

Figure S2 (a) C 1s, (b) O 1s and (c) N 1s spectra of Co_xNi_yS.

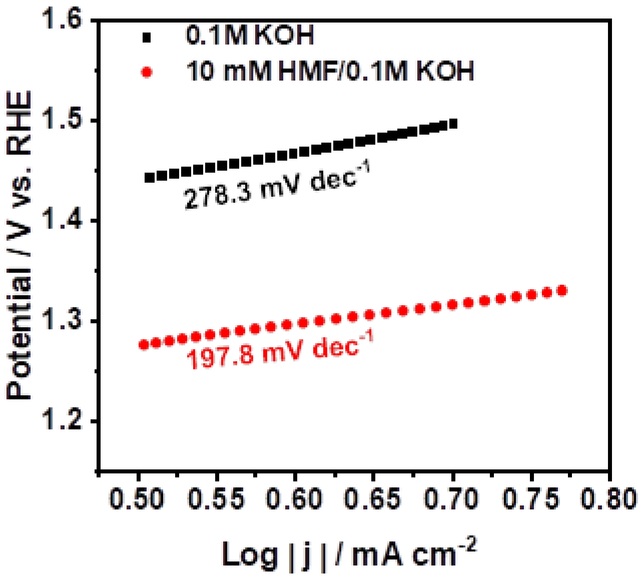

Figure S3 Tafel plots of Co_2NiS in 0.1 M KOH with and without 10 mM HMF at a scan rate of 10 mV s^{-1}.

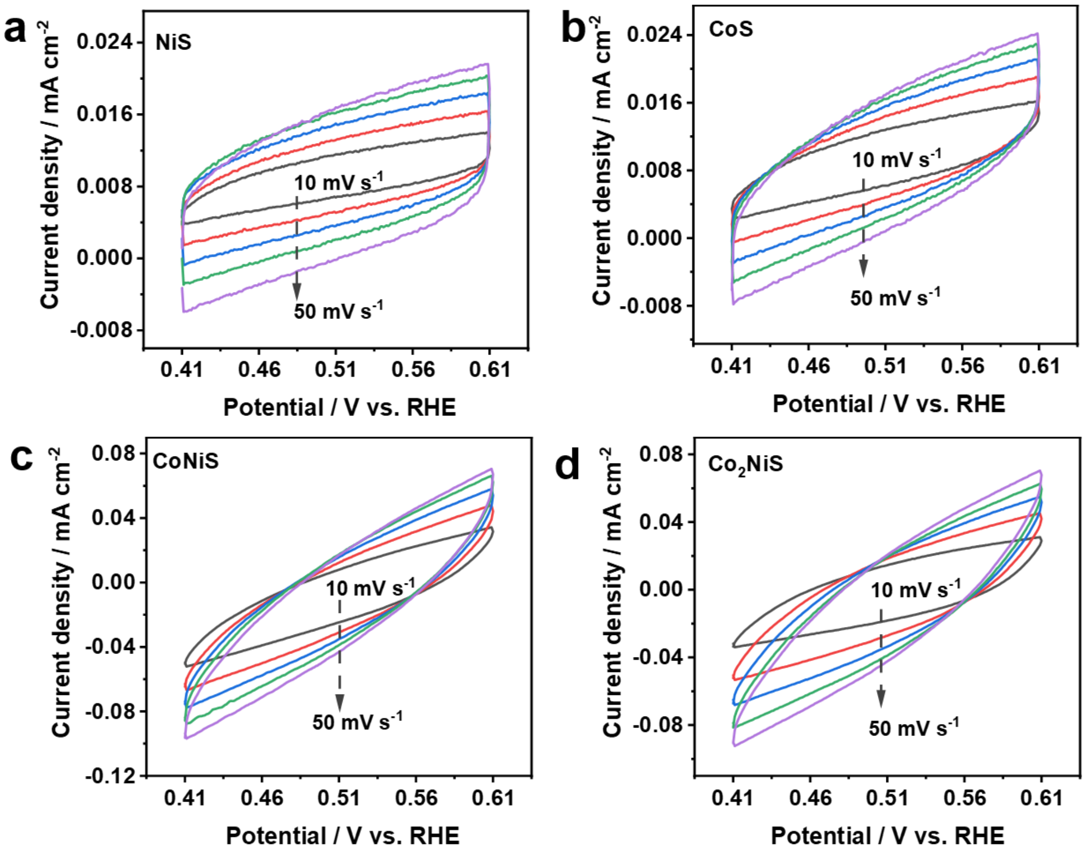

Figure S4 CV of (a) NiS, (b) CoS, (c) CoNiS, and (d) Co_2NiS in 0.1M KOH at various scan rates for estimation of double-layer capacitance.

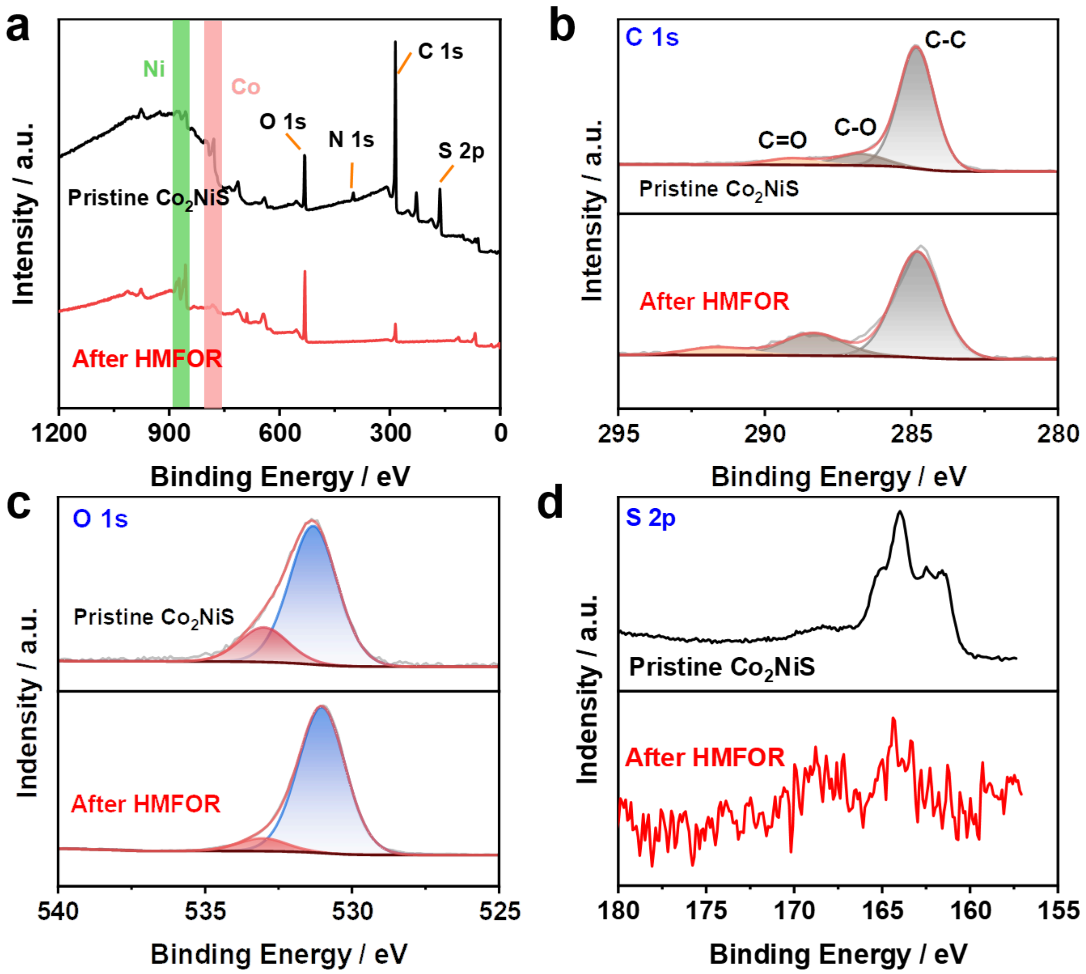

Figure S5 (a) The XPS survey spectra, (b) C 1s, (c) O 1s and (d) S 2p spectra of Co_2NiS before and after HMFOR.

Table S1 Comparison of HMF oxidation performance in literature

Catalyst	Electrolyte/ HMF concentration (mM)	Reaction time (h)	E (V vs RHE)	HMF Conversion %	FDCA Yields %	FE%	Ref
Pd_1Au_2/C	0.1 M KOH/20	1	0.90	100	83	-	1
Ni/CP	0.1 M KOH/5	44	1.36	99.7	99.4	-	2
Cu foam	0.1 M KOH/5	41	1.62	99.9	96.4	95.3	3
CoAl LDH	0.1 M KOH/10	59.1	1.52	-	-	99.4	4
NiOOH	0.1 M KOH/5	4.7	1.47	99.8	96	96	5
NiOOH	0.1 M KOH/5	1.9	1.56	96.8	86.6	86.6	5
NiOOH	0.1 M KOH/5	1.1	1.62	88.0	69.9	69.9	5
NiOOH	0.1 M KOH/5	0.8	1.71	80.3	53.5	53.5	5
CoOOH	0.1 M KOH/5	22	1.56	95.5	35.1	35.1	5
CoOOH	0.1 M KOH/5	4.6	1.62	87.5	25.6	25.6	5
CoOOH	0.1 M KOH/5	1.7	1.71	48.8	6.02	6.02	5
FeOOH	0.1 M KOH/5	2.3	1.71	16	1.6	1.6	5
NiCoBDC-NF	0.1 M KOH/10	4	1.55	-	58	-	6
Co_2NiS	0.1 M KOH/10	8	1.45	84.5	54.0	59.2	This work

References

1. Chadderdon, D. J.; Xin, L.; Qi, J.; Qiu, Y.; Krishna, P.; More, K. L.; Li, W., Electrocatalytic oxidation of 5-hydroxymethylfurfural to 2,5-furandicarboxylic acid on supported Au and Pd bimetallic nanoparticles. *Green Chemistry* **2014,** *16* (8), 3778-3786.

2. Lu, X.; Wu, K.-H.; Zhang, B.; Chen, J.; Li, F.; Su, B.-J.; Yan, P.; Chen, J.-M.; Qi, W., Highly Efficient Electro-reforming of 5-Hydroxymethylfurfural on Vertically Oriented Nickel Nanosheet/Carbon Hybrid Catalysts: Structure–Function Relationships. *Angewandte Chemie International Edition* **2021,** *60* (26), 14528-14535.

3. Nam, D.-H.; Taitt, B. J.; Choi, K.-S., Copper-Based Catalytic Anodes To Produce 2,5-Furandicarboxylic Acid, a Biomass-Derived Alternative to Terephthalic Acid. *ACS Catalysis* **2018,** *8* (2), 1197-1206.

4. Song, Y.; Li, Z.; Fan, K.; Ren, Z.; Xie, W.; Yang, Y.; Shao, M.; Wei, M., Ultrathin layered double hydroxides nanosheets array towards efficient electrooxidation of 5-hydroxymethylfurfural coupled with hydrogen generation. *Applied Catalysis B: Environmental* **2021,** *299*, 120669.

5. Taitt, B. J.; Nam, D.-H.; Choi, K.-S., A Comparative Study of Nickel, Cobalt, and Iron Oxyhydroxide Anodes for the Electrochemical Oxidation of 5-Hydroxymethylfurfural to 2,5-Furandicarboxylic Acid. *ACS Catalysis* **2019,** *9* (1), 660-670.

6. Cai, M.; Zhang, Y.; Zhao, Y.; Liu, Q.; Li, Y.; Li, G., Two-dimensional metal–organic framework nanosheets for highly efficient electrocatalytic biomass 5-(hydroxymethyl)furfural (HMF) valorization. *Journal of Materials Chemistry A* **2020,** *8* (39), 20386-20392.
